# Ferritin Iron Mineralisation: Route of Fe^3+^ Transfer From the Ferroxidase Centre to the Inner Cavity of Human H‐Chain Ferritin

**DOI:** 10.1002/anie.1203843

**Published:** 2026-05-30

**Authors:** Zinnia Bugg, Justin M. Bradley, Andrew M. Hemmings, Nick E. Le Brun

**Affiliations:** ^1^ Centre For Molecular and Structural Biochemistry School of Chemistry Pharmacy and Pharmacology University of East Anglia Norwich UK; ^2^ Current Address: Institut für Biochemie Albert‐Ludwigs‐Universität Freiburg Freiburg Germany; ^3^ Centre For Molecular and Structural Biochemistry School of Biological Sciences University of East Anglia Norwich UK; ^4^ International Research Centre For Food and Health College of Food Science and Technology Shanghai Ocean University Nanhui New City Shanghai China

**Keywords:** ferritin, iron, transport, oxidation, storage

## Abstract

Ferritin‐catalysed Fe^2+^ oxidation by reaction with O_2_ occurs at an intra‐subunit diiron site known as the ferroxidase centre (FoC). Currently, how Fe^3+^, the key substrate for iron core nucleation/mineralisation, transfers from the FoC to the inner protein surface/central cavity where the mineral is laid down is unknown. Iron‐binding sites that become occupied following exposure of anaerobic, Fe^2+^‐bound human cytosolic H‐chain ferritin (HuHF) to O_2_ were identified by time‐resolved x‐ray crystallography. In addition to the two FoC iron sites, three further sites were identified, each involving Glu61 as a coordinating residue. Substitution by a non‐coordinating residue (variant E61A) eliminated binding at these additional iron sites. Solution kinetic studies of Fe^2+^ oxidation and iron core mineralisation in wild‐type HuHF and its E61A variant showed that rapid Fe^2+^ oxidation was unaffected by loss of Glu61, ruling out an important role for these sites in either guiding Fe^2+^ to the FoC, or in the mechanism of FoC‐catalysed Fe^2+^ oxidation. Conversely, the transfer of Fe^3+^ out of the FoC and core mineralisation were both severely affected in the E61A variant. A mechanism for Fe^3+^ transfer from the FoC to the inner protein surface is proposed.

## Graphical abstract

Proposed mechanism for Fe^3+^ transport from the FoC (sites 1 and 2) of human H‐chain ferritin following oxidation of the di‐Fe^2+^ form. Glu61, aided by the flexibility of its sidechain, plays a key role in coordinating Fe^3+^ at three sites (3, 4 and 5) that mark out the transit route.

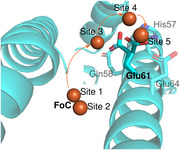

## 1 Introduction

The essential but potentially highly toxic micronutrient iron must be carefully managed in cells [1, 2]. Ferritins, which are key players in cellular iron metabolism, are in general composed of 24 subunits that spontaneously assemble to form a macromolecule of 4‐, 3‐, 2‐ symmetry (formally a rhombic dodecahedron) that can be thought of as a football‐like cage protein [3, 4]. Ferritins function by catalysing the oxidation of excess Fe^2+^ and the formation of a Fe^3+^‐oxyhydroxide mineral that is solubilised within the central cavity of the protein. This enables cells to retain iron in an unreactive (and thus safe) form that can be subsequently accessed if demand outstrips cellular iron supply. Therefore, ferritins function in both iron storage and iron detoxification with their principal function depending on the particular protein/cell [1].

**FIGURE 1:**
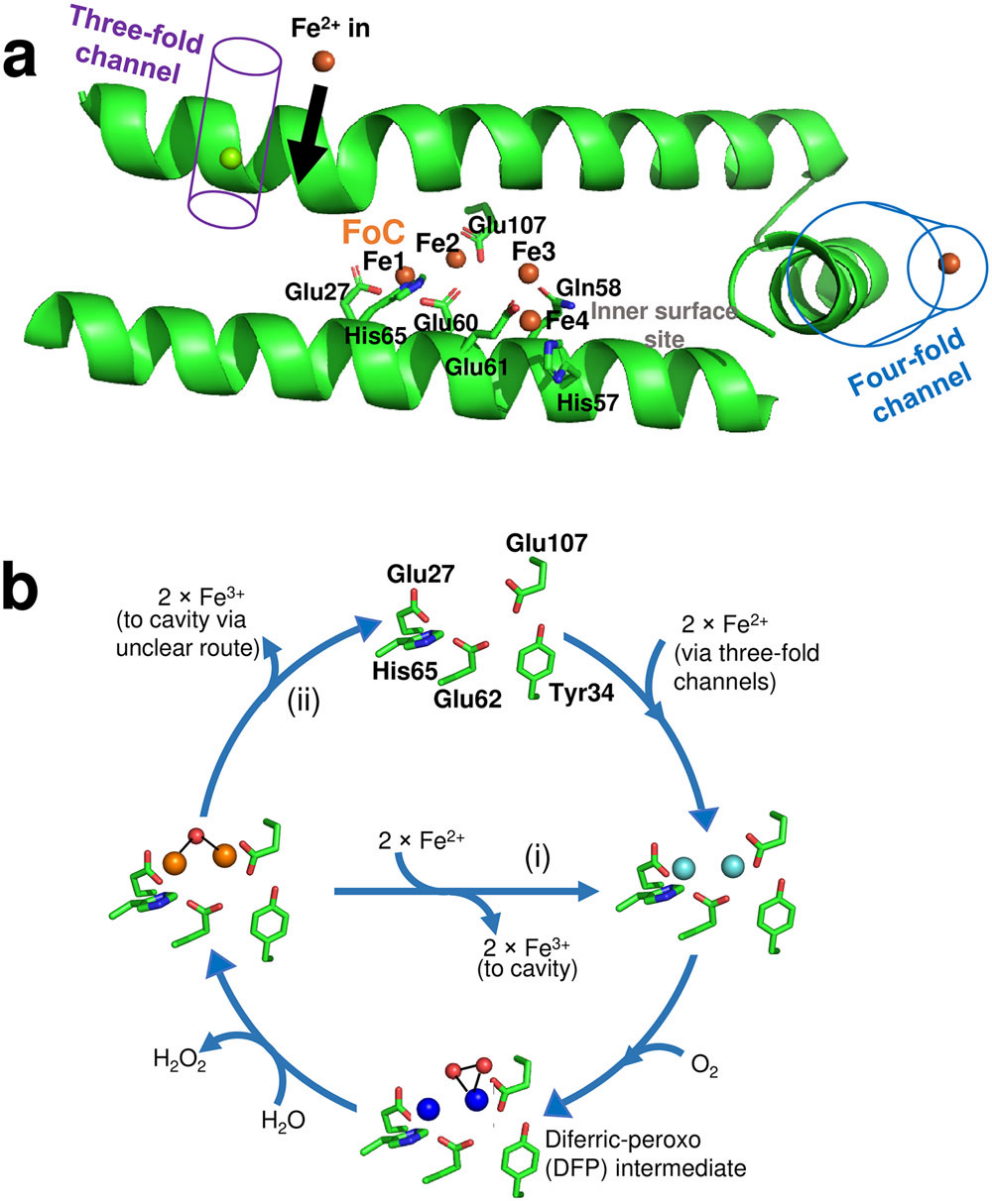
Route of Fe^2+^ entry into the protein and to the FoC and mechanism of Fe^2+^ oxidation in HuHF. (a) Structure of the HuHF subunit showing the route of Fe^2+^ through the three‐fold channel towards the diiron FoC. Additional, near‐FoC iron sites Fe3 an Fe4 are shown. (b) Schematic representation of the mechanism of Fe^2+^ oxidation catalysed by HuHF. Starting at the top, two equivalents of Fe^2+^ traverse the protein coat via a three‐fold channel and bind at a vacant (apo) FoC to give di‐Fe^2+^ FoC (right). Oxidation of each Fe^2+^ with transfer of the resulting electrons onto O_2_ yields a di‐Fe^3+^ peroxo intermediate (bottom). This is hydrolysed to give a metastable di‐Fe^3+^ oxo species and peroxide (left). In pathway (i) Fe^3+^ is displaced by incoming Fe^2+^ substrate, regenerating the di‐Fe^2+^ form of the FoC. Displaced Fe^3+^ is eventually incorporated into the growing ferric oxyhydroxide mineral core. In the absence of further Fe^2+^ substrate (pathway (ii)), the apo form of the FoC is regenerated by the slower exit of Fe^3+^ from the FoC into the interior of the protein where it also contributes to the accumulation of a mineral core. Fe^2+^ ions are indicated in cyan, Fe^3+^ are in brown, and the DFP is in royal blue.

Mammalian cytosolic ferritins are composed of two subunit types, H‐chain and L‐chain, which are isostructural and can therefore co‐assemble in any ratio. Indeed, the precise subunit composition of cytosolic ferritins depends on which tissue the ferritin is localised to, with H‐chain the dominant subunit in heart ferritin, and L‐chain dominant in spleen and liver ferritins [3]. Catalysis of Fe^2+^ oxidation occurs at an intra‐subunit diiron site, known as the ferroxidase centre (FoC), that is found only in the H‐chain subunit [5]. Formation of the mineral iron core, which depends on a supply of Fe^3+^ from FoC activity, is initiated on the inner surface of the protein cavity, with the nucleation site localised to the L‐chain subunit [6]. In brief, the mechanism by which ferritins generate an iron core involves uptake of Fe^2+^ into the protein via hydrophilic channels at the 3‐fold axes, which is guided to the diiron FoCs to generate the di‐Fe^2+^ form. Oxidation of the pair of Fe^2+^ ions occurs through reaction with O_2_, resulting in H_2_O_2_ and a metastable di‐Fe^3+^ form. Fe^3+^ then exits the FoC and enters the central cavity where it is incorporated into the growing mineral (Figure 1). The cycle continues until Fe^2+^ is no longer in excess of cellular requirement [3, 5]. The mechanism of Fe^3+^ exit from the FoC is, as yet, unclear.

Studies of animal ferritins over the past 40 years have largely focused on homopolymers generated from recombinant DNA technologies. Ferritins, such as the H’‐chain of *Rana catesbeiana* (also known as frog M‐chain) and the H‐chain of human ferritin (HuHF), have been intensively studied [3, 7]. In contrast to many prokaryotic ferritins, for which crystals of protein with iron bound are readily obtained [8, 9, 10, 11], structures of animal ferritins with iron bound at their FoCs have proved much harder to obtain. While these proteins readily crystallise, the conditions required for this include high pH (typically pH 9) and high concentration (>molar) of Mg^2+^ salts as precipitant. High pH limits the solubility of Fe^2+^ [12] and promotes its oxidation to Fe^3+^ [13], and high concentrations of Mg^2+^ ions effectively compete for Fe^2+^‐binding sites in the protein [14]. Consequently, it was not until 2012 when the first iron‐bound animal ferritin structure was published [15]. Improvements in resolution came through the development of a novel method for iron‐enrichment of protein crystals involving their exposure to solid ferrous ammonium sulfate (Mohr's salt), rather than soaking them in a solution of Fe^2+^. This reportedly resulted in increased local concentration of Fe^2+^ able to out‐compete Mg^2+^ for Fe^2+^‐binding sites on the protein [14, 16]. Increasing the period of exposure to Fe^2+^ enabled the observation in Frog H’ and HuHF of additional iron‐binding sites, termed Fe3 and Fe4 (named in sequence in terms of proximity to the FoC Fe1 and Fe2 sites), outside of the FoCs. Fe3 involves coordination by Gln58 and Glu61 and Fe4 by His57 and Glu61 [16]. Binding of iron at these sites appeared to mark out a route for Fe^2+^ as it exits the three‐fold channel and transfers to the FoC.

More recently, high‐resolution structures of human mitochondrial ferritin (FtMt) were reported [17, 18]. This protein is unusual amongst animal ferritins in that it is naturally a homopolymer of subunits that are ∼80% identical to the cytosolic H‐chain subunit. The most recent study employed a novel crystallisation strategy to observe iron‐bound FtMt in which protein crystals were grown anaerobically from solutions containing Fe^2+^ as well as Mg^2+^ [18]. In addition to iron bound at the two FoC sites, a further iron site was observed, involving residues His57 and Glu61 at a site equivalent to Fe4 in the earlier HuHF studies, supporting a role for Fe4 in guiding Fe^2+^ to the FoC.

A consequence of FtMt being a homopolymer is that both the FoC and the mineral core nucleation site must be present within the same subunit. Crystals were subsequently exposed to O_2_, resulting in turnover of FoCs and the observation of changes in iron binding that provided important mechanistic insight, including the first observation of the nascent iron mineral as a pentanuclear ferric‐oxo cluster involving sites Fe4’, Fe5, Fe6, Fe7 and Fe8 on the inner surface of a ferritin containing H‐chain‐type subunits. Residues His57, Glu61 and Glu64 were found to be interacting with the nascent ferric‐oxo cluster. Two of these residues, His57 and Glu61, are ligands to Fe4 (observed prior to the introduction of O_2_) and also to Fe4’, which is a modified version of Fe4 observed after introduction of O_2_. Thus, following mineral core nucleation, site Fe4 is no longer available to bind incoming Fe^2+^ as its protein ligands are already involved in iron binding. Furthermore, solution kinetics of site‐directed variants of the associated residues pinpointed Glu61 to be not only crucial for mineral nucleation but also for the transfer of Fe^3+^ from the FoC, leading to regeneration of the apo form of the FoC. These data also showed that Glu61 and Glu64 (but not His57) influence iron binding to the FoC, postulated to be a result of contributions of their negative charges to the electrostatic field that guides Fe^2+^ ions towards the active site, rather than as ligands to Fe^2+^ as part of stable transit site complexes.

Glu61 is conserved in HuHF and, as described above, is important for binding of iron at near‐FoC sites [16], leading to the proposal that Glu61 plays a role in coordinating Fe^2+^ at a transit site en route to the FoC. Earlier kinetic studies of a E61A/E64A/E67A HuHF triple variant indicated that iron core mineralisation was impacted [19], suggesting that these residues are important for mineralisation. However, a later study of a E64A/E67A variant did not support an important role for Glu64 or Glu67 [20]. The previous literature, together with the key role played by Glu61 in FtMt [18], indicated that further investigation of the role of Glu61 in HuHF is timely. Here, we present x‐ray crystallographic and solution kinetic studies of HuHF and its E61A variant, permitting direct comparison of the consequences of the E61A substitution in HuHF and FtMt. The data clearly resolve the function of Glu61 in promoting the transfer of Fe^3+^ out of the FoC following its formation through reaction of the di‐Fe^2+^ form with O_2_.

## 2 Results and Discussion

### 2.1 High‐Resolution Structure of anaerobic Fe^2+^‐bound HuHF

**FIGURE 2:**
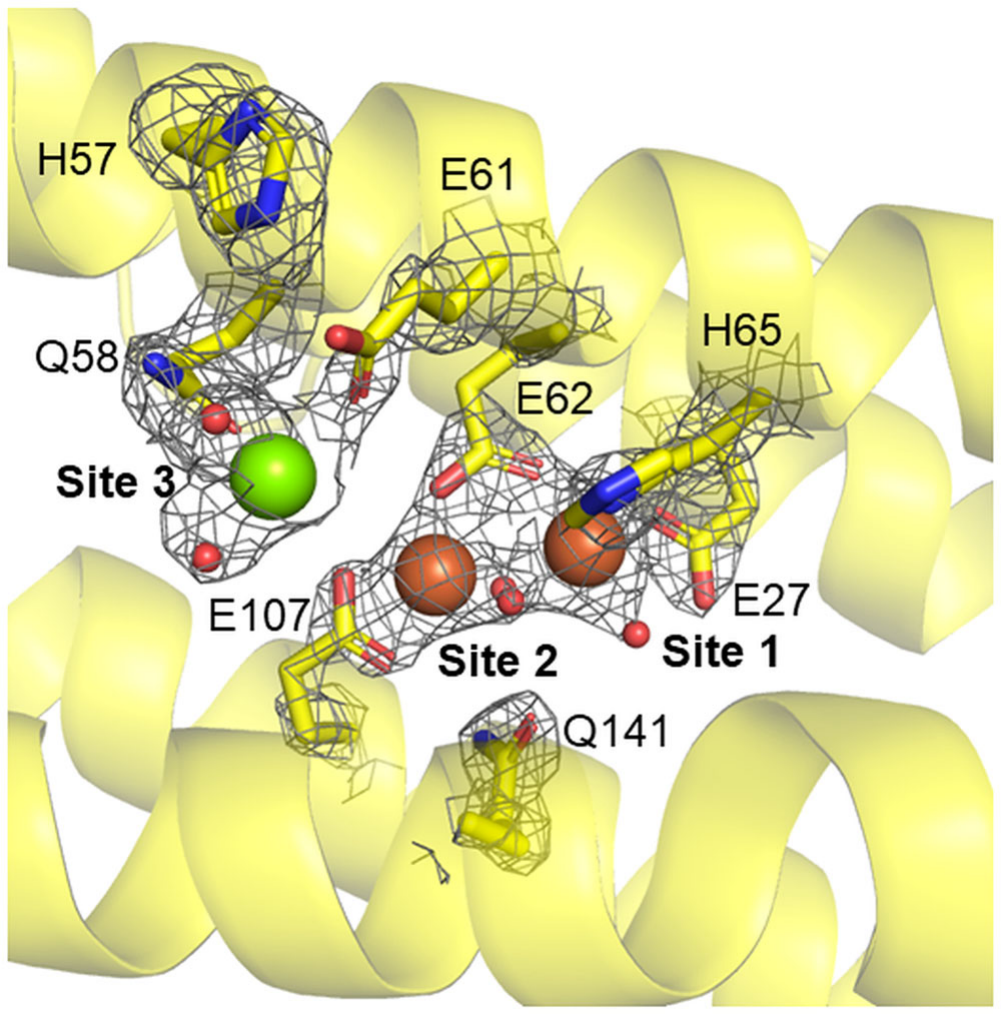
Crystal structure of the FoC area of anaerobic iron‐loaded HuHF. Fe^2+^ ions are bound at sites Fe1 and Fe2 (orange spheres) and a Mg^2+^ ion at site Fe3 (green sphere). Red spheres show water placements. The Sigma‐A weighted Fourier (2mFo‐DFc) map contoured at 1 σ for ions and associated residues is shown in grey mesh. Images made in PyMOL with data collected in this report. We note that, consistent with the data reported here, the structure of HuHF derived from anaerobic crystals of HuHF exposed to Mohr's salt for 8 min was described by Pozzi and co‐workers [16], with Fe^2+^ bound at sites Fe1 and Fe2 and no other metals bound at near‐FoC sites.

Iron‐enriched crystals of HuHF were generated using the methodology described above for FtMt, in which ferritin was anaerobically co‐crystalised with Fe^2+^ from drops containing 60 mM ferrous chloride (see Materials and Methods). The anaerobic iron‐loaded HuHF structure featured Fe^2+^ bound only at the FoC (Figure 2) and the four‐fold channel (Figure S1). Site Fe1 was almost at full occupancy, while Fe2 was somewhat lower, as often observed (fractional occupancies of the FoC metal sites are given in Table 1), with an Fe─Fe distance of 3.3 Å. Mg^2+^ was bound at a site previously characterised as Fe3 (closest of the additional sites to the FoC) following exposure of aerobically grown crystals of HuHF to Mohr's salt. There was no anomalous scattering at the three‐fold channel, where density was therefore modelled as hexa‐aqua Mg^2+^, Figure S2. This reflects the competition between Mg^2+^ and Fe^2+^ ions present in the drop solution for stable binding at the three‐fold channel site. This may inhibit Fe^2+^ access to the FoC, but clearly does not prevent it over the course of the crystallisation.

**TABLE 1:** Fractional occupancies of iron sites and Fe1─Fe2 distances in wild‐type HuHF and E61A HuHF structures with previously published data for iron‐loaded HuHF structures (shaded in grey) (pdb codes 4Y08 [1 min], 4ZJK [5 min], 4YKH [30 min]) included for comparison [16]. We note that rates of processes in the crystal occur much more slowly than in solution; hence the extended crystal soak times.

	Iron sites					
	FoC sites			Near‐FoC sites		
	Occupancies			Occupancies		
Protein	Fe1	Fe2	Fe‐Fe distance (Å)	Fe3	Fe4	Fe5
HuHF anaerobic	0.9	0.65	3.30	—	—	—
HuHF 2‐min O_2_ soak	0.8	0.6	3.25	0.3	0.3	—
HuHF 20‐min O_2_ soak	1.0	0.9	3.40	—	0.5	—
HuHF 3‐h O_2_ soak	1.0	0.9	3.34	0.3	0.3	0.2
E61A HuHF anaerobic	1.0	0.8	3.41	—	—	—
E61A HuHF 1‐h O_2_ soak	1.0	0.8	3.27	—	—	—
HuHF 1‐min Fe exposure	0.7	0.2	3.47	0.3	0.2	—
HuHF 5‐min Fe exposure	1.0	0.6	3.48	0.5	0.4	—
HuHF 30‐min Fe exposure	1.0	0.4	3.52	0.5	0.3	—

Although not shown, nor deposited in the pdb, Pozzi et al. [16] described a high‐resolution structure of apo HuHF derived from anaerobically grown crystals exposed to Mohr's salt for 8 min. This revealed Fe^2+^ bound only at Fe1 and Fe2 sites, with fractional occupancies of about 0.8 for Fe1 and 0.3 for Fe2 and a Fe1─Fe2 distance of 3.52 Å with one water/hydroxide molecule symmetrically bridging the two irons. The lower occupancies for sites Fe1 and Fe2 resulting from exposure to an iron salt compared to those for HuHF co‐crystalised with Fe^2+^ reflect the restricted Fe^2+^ transport through the crystal. Importantly, both structures are consistent in that they provide no evidence for Fe^2+^ binding at a third site close to the FoC prior to the introduction of O_2_.

### 2.2 Time‐Dependent Structural Analysis of HuHF‐Catalysed Fe^2+^ Oxidation by Exposure to O_2_

**FIGURE 3:**
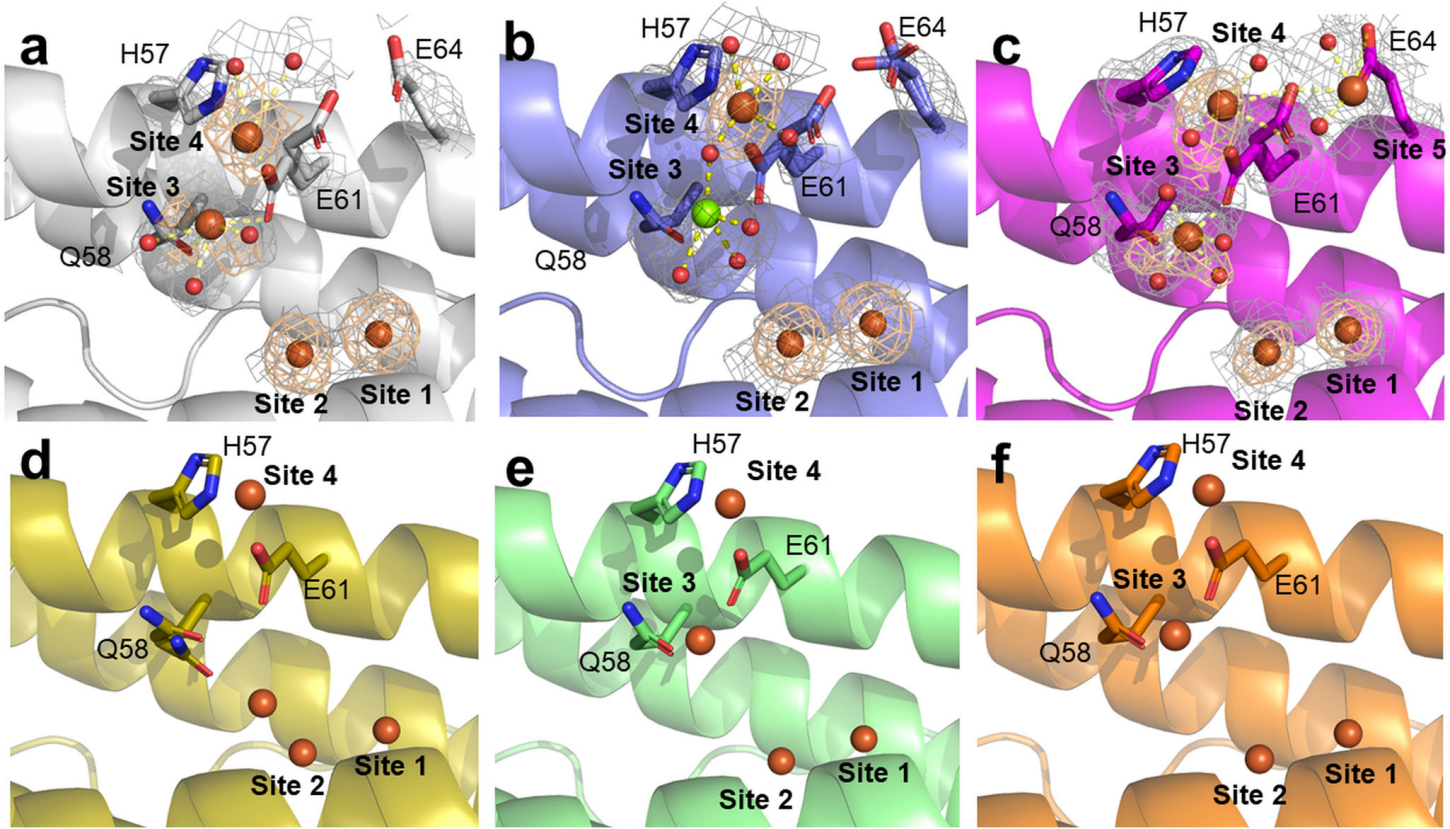
Crystal structures of iron‐loaded HuHF. Structures are those following varying O_2_‐soak periods of (a) 2‐min, (b) 20‐min and (c) 3‐h, with iron ions as orange spheres and sites 1–5 (Fe1‐5) and associated residues labelled. Published structures of iron‐bound HuHF following varying iron soak times of (d) 1‐min, (e) 5‐min and (f) 30‐min. Grey mesh shows the Sigma‐A weighted Fourier (2mFo‐DFc) map contoured at 1 σ for ions and the associated residues for the iron sites. Orange mesh shows the anomalous scattering map collected at the iron K‐edge contoured to 4σ. Red spheres show water placement. Images made in PyMOL using data collected in this project and 4Y08 (d), 4ZJK (e) and 4YKH (f) [16].

In addition to the anaerobic HuHF structure above, other structures were obtained from crystals of HuHF exposed to O_2_ for 2 min, 20 min and 3 h. Iron‐binding sites observed on the inner surface of the protein cage are shown in Figure 3a–c. There were no significant changes in fractional occupancies of the FoC sites nor in the Fe1─Fe2 distance after the 2‐min O_2_ soak, see Table 1. The fractional occupancies of the FoC sites in previously published structures (Figure 3d,e) varied significantly with iron soak time, likely an effect of iron entry and transfer being more restricted in solid‐to‐solid diffusion. In the 2‐min O_2_ soak structure, iron was also observed at additional sites close to the FoC, equivalent to sites Fe3 (coordinated by Gln58 and Glu61) and Fe4 (coordinated by His57 and Glu61), see Figure 3a, as previously described for HuHF [16]. Fe3 and Fe4 each had an occupancy of ∼0.3 (Table 1).

After 20‐min O_2_ soak, FoC sites Fe1 and Fe2 were full or almost full, while Fe4 was at an occupancy of ∼0.5. Surprisingly, Fe3 was no longer occupied (Figure 3b). After 3‐h O_2_ soak, occupancies of Fe1 and Fe2 were unchanged, while those of sites Fe3 and Fe4 were similar to the 2‐min O_2_ soak (Table 1). This structure also featured an additional iron site (Fe5) on the inner surface coordinated by Glu61 and Glu64 (Figure 3c, Table 1).

A site equivalent to Fe5 was previously observed for FtMt following a 20‐min O_2_ soak [18]. However, in that case, it was accompanied by four additional Fe ions in the form of a ferric‐oxo cluster, representing the nascent iron mineral on the inner surface of FtMt. In the case of HuHF, observation of anomalous scattering at site Fe5 required the O_2_ soaking time to be extended to 3 h and, even at this extended soaking time, there was no sign of additional electron density protruding into the interior cavity, consistent with the physiologically relevant nucleation site for the mineral core being located on the inner surface of the L‐chain subunit HuLF [6]. Nevertheless, even though homopolymeric HuHF is not a physiologically relevant form of the protein, in vitro studies have demonstrated its capacity to generate an iron mineral. The observation of iron at site Fe5 after a 3‐h O_2_ soak time might indicate that this site plays a role in facilitating transfer of Fe^3+^ for nucleation in the HuHF homopolymer. We note that Tb^3+^ bound at a site equivalent to Fe5 was reported following very early structural studies, leading to the suggestion that this site could represent a Fe^3+^‐binding site that is occupied following oxidation at the FoC [21].

### 2.3 Glu61 is Important for the Observation of Fe^3+^ at Near‐FoC Sites Following Oxidation of Fe^2+^ at the FoC

**FIGURE 4:**
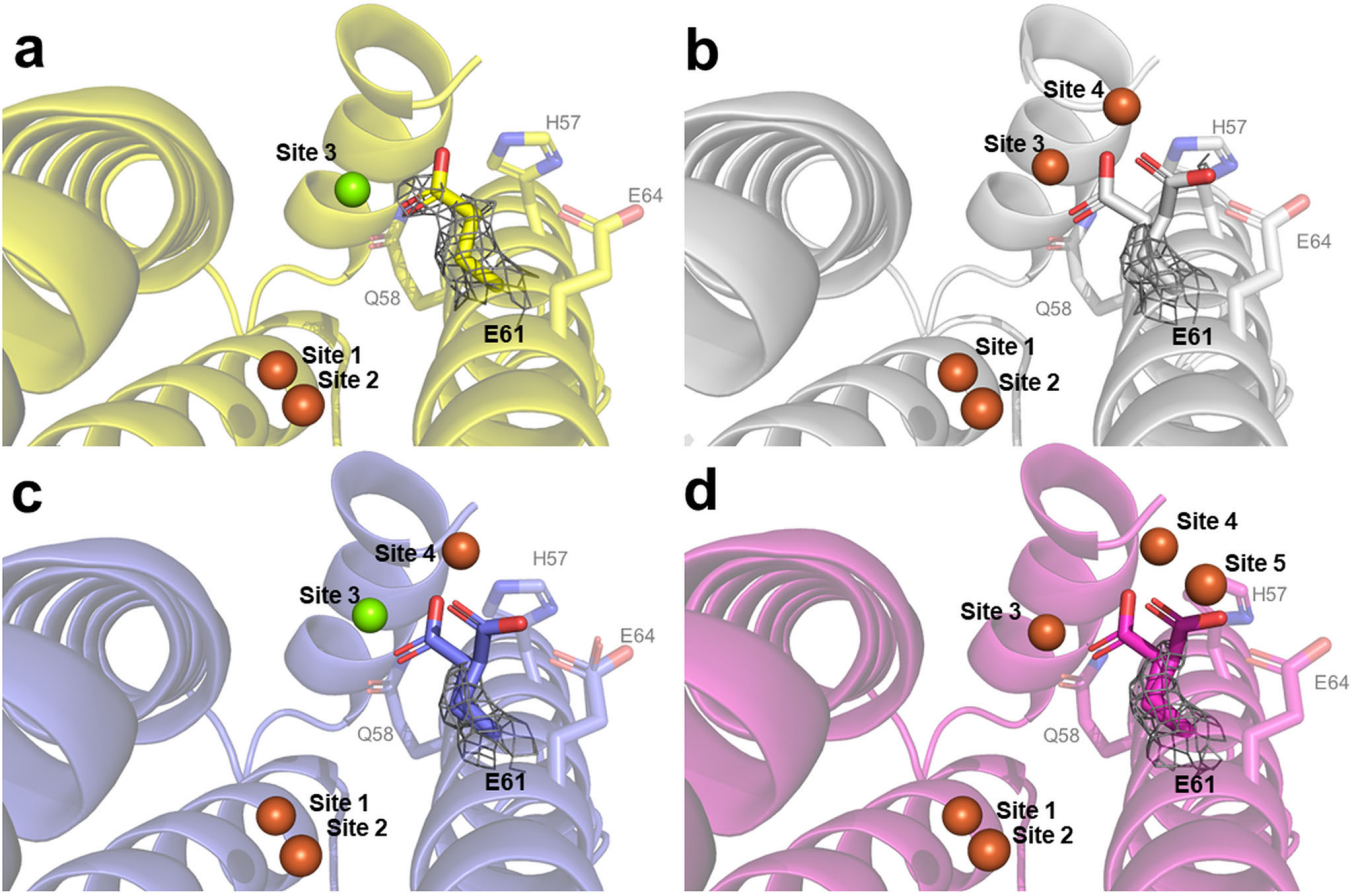
Flexibility of the Glu61 sidechain during mineralisation of HuHF. Crystal structures of iron‐loaded HuHF with O_2_‐exposure times of (a) 0‐min, (b) 2‐min, (c) 20‐min, and (d) 3‐h, with images centred on the Glu61 residue. Grey mesh shows the Sigma‐A weighted Fourier (2mFo‐DFc) map contoured at 1 σ for the Glu61 sidechain. Iron and magnesium atoms shown by orange and green spheres, respectively. Images made in PyMOL.

The positioning of the sidechain of residue Glu61 varied upon exposure to O_2_. In the anaerobic HuHF structure (Figure 4a), the Glu61 sidechain pointed towards the active site. Following O_2_ exposure, the density was no longer consistent with a single rotamer orientation of Glu61, instead indicating two orientations, one as before, pointing towards the active site, and the other pointing towards the inner surface. Further evidence of two conformations of the Glu61 side chain is apparent from Fo–Fc maps shown in Figure S3, in which unmodelled positive and negative density are observed when the second conformation is not included in the model. These two orientations were observed with a 50:50 occupancy of each rotamer for all O_2_‐exposure times, suggesting an equilibrium distribution between two orientations as iron is shuttled between the FoC and the interior of the protein.

**FIGURE 5:**
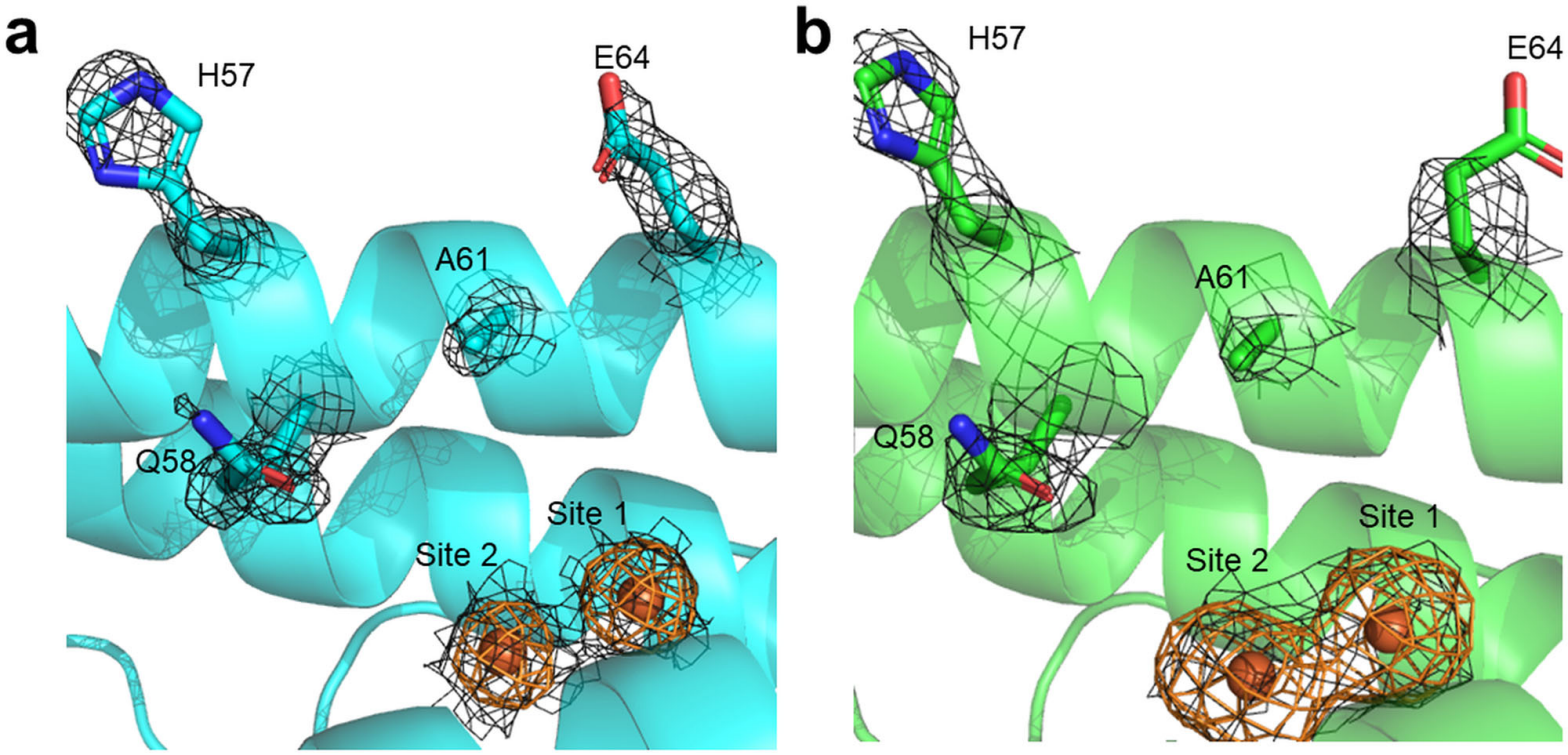
Crystal structures of iron‐loaded E61A HuHF. Structures shown are those following (a) 0‐min and (b) 1‐h O_2_ soaks. Iron sites (orange spheres) of the FoC and residues identified as being associated with iron sites 3, 4 and 5 are indicated. Grey mesh shows the Sigma‐A weighted Fourier (2mFo‐DFc) map contoured at 1 σ for ions and the associated residues for the iron sites. Orange mesh shows the anomalous scattering map collected at the iron K‐edge contoured at 4σ. Images made with PyMOL.

Given the importance of Glu61 in coordinating iron in the structures above, a variant of HuHF was generated in which Glu61 was replaced by alanine. Crystals of the E61A variant were grown in the same way as for the wild‐type protein (co‐crystallised with Fe^2+^ under anaerobic conditions). The resulting structure of E61A HuHF featured Fe^2+^ bound at the FoC sites Fe1 and Fe2, with a Fe─Fe distance similar to that observed for the wild‐type protein and an occupancy of site Fe2 somewhat higher (Table 1). Following exposure of crystals to O_2_ for 1 h, occupancies and Fe1─Fe2 distances were not significantly different. Importantly, the structure did not feature any metal binding at sites Fe3, Fe4 or Fe5, Figure 5. This indicated that Glu61 is important for Fe^3+^ binding at these near‐FoC sites that only become occupied in the wild‐type protein following reaction with O_2_.

The requirement for crystals to be exposed to O_2_ to observe anomalous scattering at sites Fe3–Fe5 suggests they contain oxidised iron following turnover of the FoC. Multiple conformations of Glu61 are consistent with a role for this residue in shuttling iron between the FoC sites and those on the inner surface of the protein. However, the structural data do not unambiguously determine the oxidation state of the iron observed bound to the protein. Therefore, to support the above interpretation, the consequences of the absence of Glu61 on the kinetics of HuHF‐catalysed Fe^2+^ oxidation were investigated by solution assays.

### 2.4 Glu61 plays a Key Role in Iron Core Mineralisation

**FIGURE 6:**
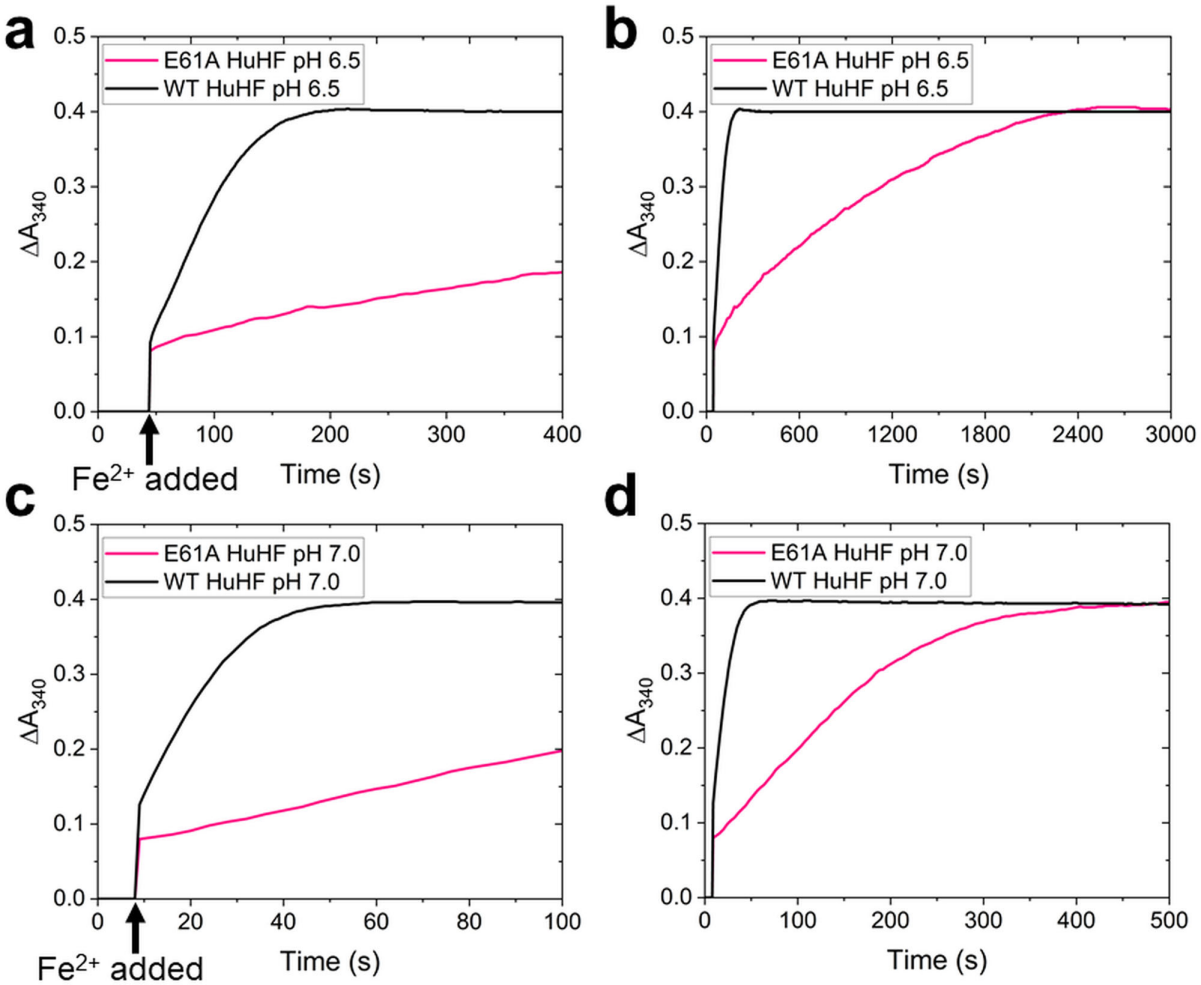
Iron mineralisation activity assays of E61A and wild‐type HuHF. Assays for 0.5 µM wild‐type (black) and E61A HuHF (pink) were recorded in both 100 mM MES pH 6.5 (a and b) and 100 mM MOPS pH 7.0 (c and d) with 200 µM ferrous ammonium sulfate in 1 mM HCl at 25°C and measured the change in absorbance at 340 nm. Plots of each assay are shown on two different timescales corresponding to the periods over which mineralisation occurred in wild‐type HuHF and E61A HuHF. Black arrows in the shorter time period plots of (a) and (c) indicate the point at which Fe^2+^ ions were added.

Ferritin activity, resulting in formation of a ferric mineral core (Figure S4), is known to be strongly pH dependent. Therefore, to connect kinetic data with prior reports conducted at pH 6.5 (FtMt [18]) and pH 7.0 (HuHF [22, 23, 24, 25]) the impact of the E61A substitution on iron oxidation and mineralisation by HuHF was determined at both pH values. The iron mineralisation activity of wild‐type and variant proteins was investigated by the addition of 400 equivalents of ferrous ammonium sulfate in 1 mM HCl to ferritin (0.5 µM in 24mer) in 100 mM MES pH 6.5 or 100 mM MOPS pH 7.0. Following the addition of Fe^2+^, there was an immediate increase in A_340 nm_, Figures 6a,c, corresponding to the rapid binding and oxidation of 2 Fe^2+^ ions at the 24 FoCs of apo ferritin, resulting in 24 di‐Fe^3+^ FoCs, which give rise to absorbance in the near UV and visible region. For E61A HuHF, the initial increase in A_340 nm_ was slightly lower in comparison to the wild‐type protein, at 90% and 60% of those observed for the wild‐type protein at pH 6.5 and pH 7.0 (Figure 6a,c), respectively.

Following the initial oxidation of Fe^2+^ at FoCs, further oxidation of Fe^2+^, which corresponds to the formation of the mineral iron core (mineralisation phase) is rate‐limited by release of Fe^3+^ from the FoC (a process that takes seconds in comparison to the milliseconds of iron oxidation) [26]. Increases in A_340 nm_ during this phase were initially linear, enabling ready determination of the initial rate of mineralisation, before plateauing (at ∼0.4 AU) as Fe^2+^ was fully consumed. For wild‐type HuHF, this slower phase occurred over 160 or 50 s at pH 6.5 or at pH 7.0, respectively. Unlike the fast oxidation phase, this slower mineralisation phase was severely affected in the E61A HuHF variant. This behaviour is similar to that observed for FtMt and E61A FtMt [18].

Figure 6a,c show the assay on the timescale corresponding to the completion of Fe^2+^ oxidation for wild‐type HuHF, which highlights the small change in absorbance observed for the E61A variant over this timescale. Figure 6b,d show data on the timescale required for completion of oxidation in the E61A HuHF variant, that is, 3000 or 500 s for pH 6.5 and 7.0, respectively. Initial mineralisation rates calculated from the linear phases further highlight the impact of the E61A substitution, with a 14‐fold decrease at pH 6.5 and a 7‐fold decrease at pH 7.0 (Table 2). The severe impact on mineralisation activity of E61A HuHF suggests that the Fe3, Fe4 and Fe5 sites observed for wild‐type HuHF are functionally important for iron mineralisation of homopolymer HuHF, or that the FoC activity in this variant is inhibited to the extent that it has become rate limiting for mineral core formation. The latter possibility was investigated by employing absorbance spectroscopy in conjunction with stopped‐flow mixing to probe Fe^2+^ oxidation at the FoCs of wild‐type and variant proteins on the millisecond timescale.

**TABLE 2:** Kinetic parameters for FoC and mineralisation reactions catalysed by wild‐type and E61A HuHF proteins.

Protein	FoC reaction second order rate constant (M^−1^ s^−1^)		Mineralisation rate (µM s^−1^)	
	pH 6.5	pH 7.0	pH 6.5	pH 7.0
HuHF	1.88 (± 0.09) × 10^5^	5.69 (±0.02) × 10^5^	1.70 ± 0.04	5.60 ± 0.05
E61A	2.72 (± 0.24) × 10^5^	6.36 (±0.10) × 10^5^	0.12 ± 0.01	0.74 ± 0.04

### 2.5 Glu61 is Not Important for the Oxidation of Fe^2+^ at the FoC

Rates of FoC‐catalysed Fe^2+^ oxidation were deduced from the increase in 340 nm absorbance following the rapid aerobic mixing of equal volumes of 1 µM protein with Fe^2+^ in various stoichiometries (see Materials and Methods). Kinetic parameters were extracted by fitting of the resulting traces to bi‐exponential functions representing rapid (r) and slow (s) phases of Fe^2+^ oxidation. The rapid phase corresponds to the oxidation of Fe^2+^ at the FoC, a process that encompasses the entry of Fe^2+^ to the protein cage, movement to the active site and reaction with O_2_. The slow phase corresponds to subsequent oxidation of Fe^2+^ at the FoC, which first requires (and is rate‐limited by) exit of Fe^3+^ from the FoC.

**FIGURE 7:**
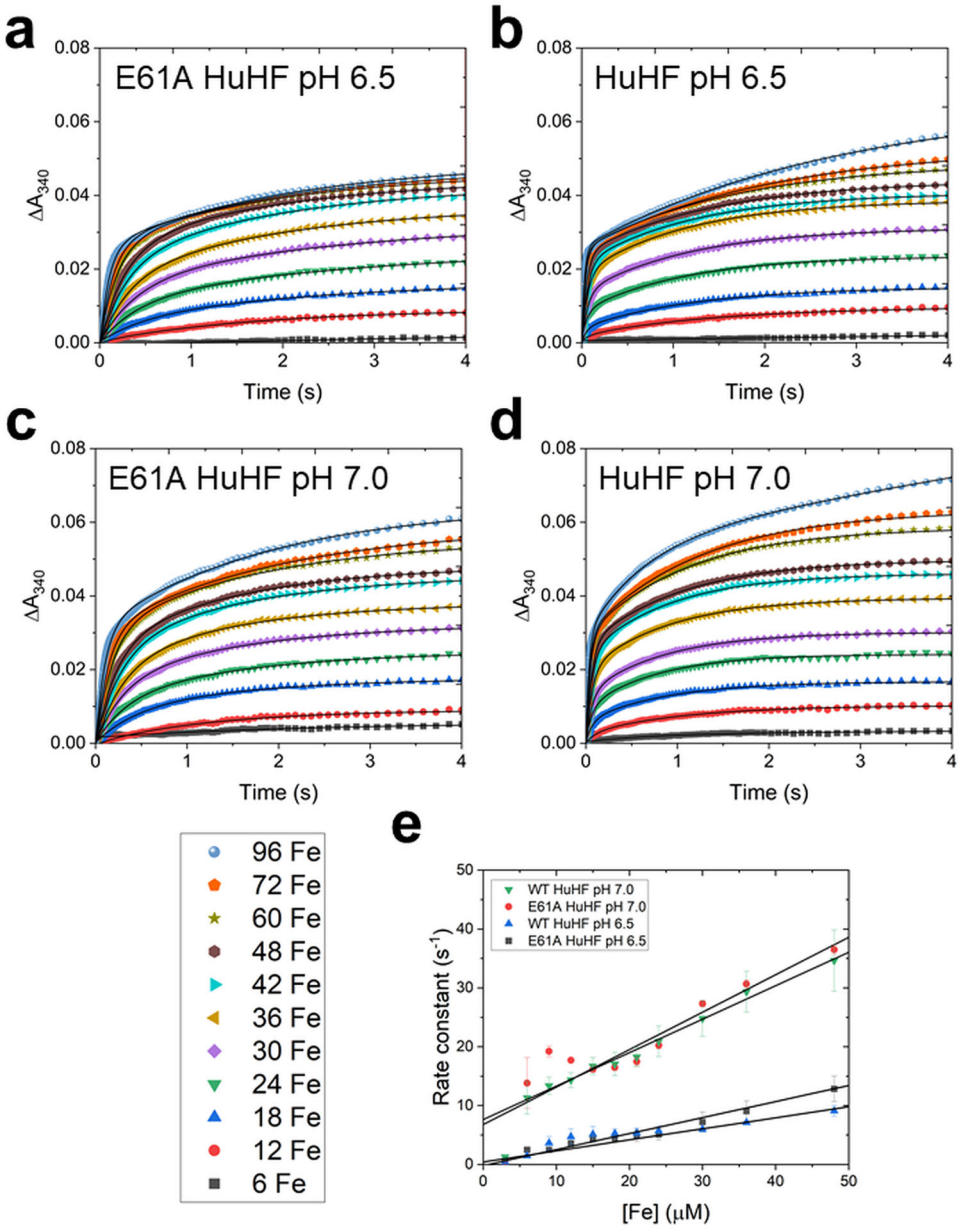
Stopped‐flow kinetic analyses of the FoC reactions of E61A and wild‐type HuHF. Titrations of 0.5 µM of E61A HuHF with 6–96 equivalents of ferrous ammonium sulfate in 1 mM HCl in (a) 100 mM MES pH 6.5 and (c) 100 mM MOPS pH 7.0. (b and d) represent equivalent data for wild‐type HuHF in 100 mM MES pH 6.5 and 100 mM MOPS pH 7.0, respectively. Changes in A_340 nm_ were measured at 25°C. Fits of the data using Equ. 1 (see Materials and Methods) are shown as solid lines. (e) Plots of rate constants derived from fitting the data for wild‐type and E61A HuHF at pH 6.5 and 7.0, as a function of Fe^2+^ concentration. Linear fits of the data gave apparent second‐order rate constants, see Table 2. Non‐zero y‐intercept, most notably at pH 7.0, is indicative of a reversible reaction.

The rapid phase catalysed by the E61A variant (Figure 7a,c) was similar to that of wild‐type HuHF (Figure 7b,d) at both pH values, indicating that activity was not inhibited in the way that iron mineralisation activity was (Figure 6). Fits of the data yielded pseudo first‐order rate constants for each addition of Fe^2+^ Figure 7, and plots of those rate constants versus Fe^2+^ concentration for E61A and wild‐type HuHF closely correspond (Figure 7e), demonstrating that the E61A substitution did not significantly affect FoC activity. For both proteins at both pH values, the pseudo first‐order rate constants varied approximately linearly with Fe^2+^ concentration. The data at pH 7 are noisy at lower Fe^2+^ concentrations, most likely because of the fast but low amplitude response. The linear dependence indicates that the rate‐limiting step is dependent on Fe^2+^, that is, that binding of Fe^2+^ at the FoC is the rate‐limiting step, such that as soon as the FoC is occupied, oxidation occurs. Therefore, the measured kinetic properties reflect Fe^2+^ binding rather than oxidation at the FoC. Interestingly, the linear fits to the pH 7 data clearly do not pass through the origin, which is characteristic of reversible binding, with the off rate constant given by the y‐intercept, in this case ∼7 s^−1^. Given that oxidation requires that both sites of the FoC are filled by Fe^2+^, one possible interpretation is that a significant off rate facilitates double occupancy of FoCs. Importantly for the current work, second‐order rate constants derived from the linear fits were similar for the two proteins, Table 2.

The slow phase (corresponding to oxidation of Fe^2+^ beyond the initial 48 Fe^2+^ per 24‐mer) was very different for wild‐type and E61A HuHF proteins (Figure 7). For the E61A variant, the amplitude associated with the slower oxidation phase measured over the first 4 s was much lower than that for the wild‐type protein (at both pH values). This represents the very beginning of the mineralisation process and so the data are consistent with the mineralisation data in Figure 6, measured over a longer period, demonstrating that the effect of substituting Glu61 is specific to events that occur after the oxidation of the first two Fe^2+^ ions per FoC.

These data indicate that Glu61 and, by association, iron sites 3, 4 and 5 are not associated with Fe^2+^ entry to the FoC of HuHF. Minimal impact on the rapid iron oxidation phase following substitution of Glu61 with a non‐coordinating ligand was also observed for FtMt [18]. However, with FtMt, the E61A substitution did affect the rate constants associated with Fe^2+^ binding/oxidation to some extent. This was concluded to be an effect of removing a negative charge, which may be important in contributing to the electrostatic potential that guides Fe^2+^ ions towards the active site. This suggested that site Fe4 may be part of the favoured pathway of entry for Fe^2+^ into FtMt. However, this can only be the case for the first Fe^2+^ ions that bind to the apo protein because once an iron‐oxo cluster is bound on the inner surface by Glu61 it is no longer available to bind incoming Fe^2+^ ions. Despite its conservation between the two protein sequences, the data do not support any role for Glu61 in Fe^2+^ entry in HuHF.

### 2.6 Glu61 is Key for the Rate at Which Fe^3+^ Exits the FoC

The rapid oxidation activity associated with the FoCs is only observed when the FoC is in the apo (i.e., vacant) form [26]. To further explore the role of Glu61, the rate at which rapid oxidation activity was recovered following oxidation of Fe^2+^ at FoC sites of wild‐type and variant E61A HuHF was investigated and compared to that observed for the apo active sites [18]. Because the rapid oxidation activity requires vacant FoCs, the recovery rate informs on the rate at which Fe^3+^ ions exit the FoC (for nucleation and growth of the mineral core) following oxidation of Fe^2+^. This provides an insight into the rate at which the ferric product of the FoC activity is cleared from the active site and incorporated into the growing mineral core.

Here, assays of the recovery of rapid oxidation activity employed HuHF samples to which 200 equivalents of Fe^2+^ were added aerobically and allowed to fully oxidise. At this point, stopped‐flow rapid mixing was used to measure the increase in A_340 nm_ over 10 s following aerobic mixing of a further 48 equivalents of Fe^2+^ added at increasingly long delay times after completion of oxidation of the initially added 200 Fe^2+^ ions. The observation of significant rapid oxidation at short incubation times would indicate fast recovery of the vacant (apo) form of the FoC, and hence, a significant rate associated with transport of Fe^3+^ out of the FoC. Observation of little rapid oxidation would indicate slow recovery and a low rate of transfer of Fe^3+^ away from the FoC.

**FIGURE 8:**
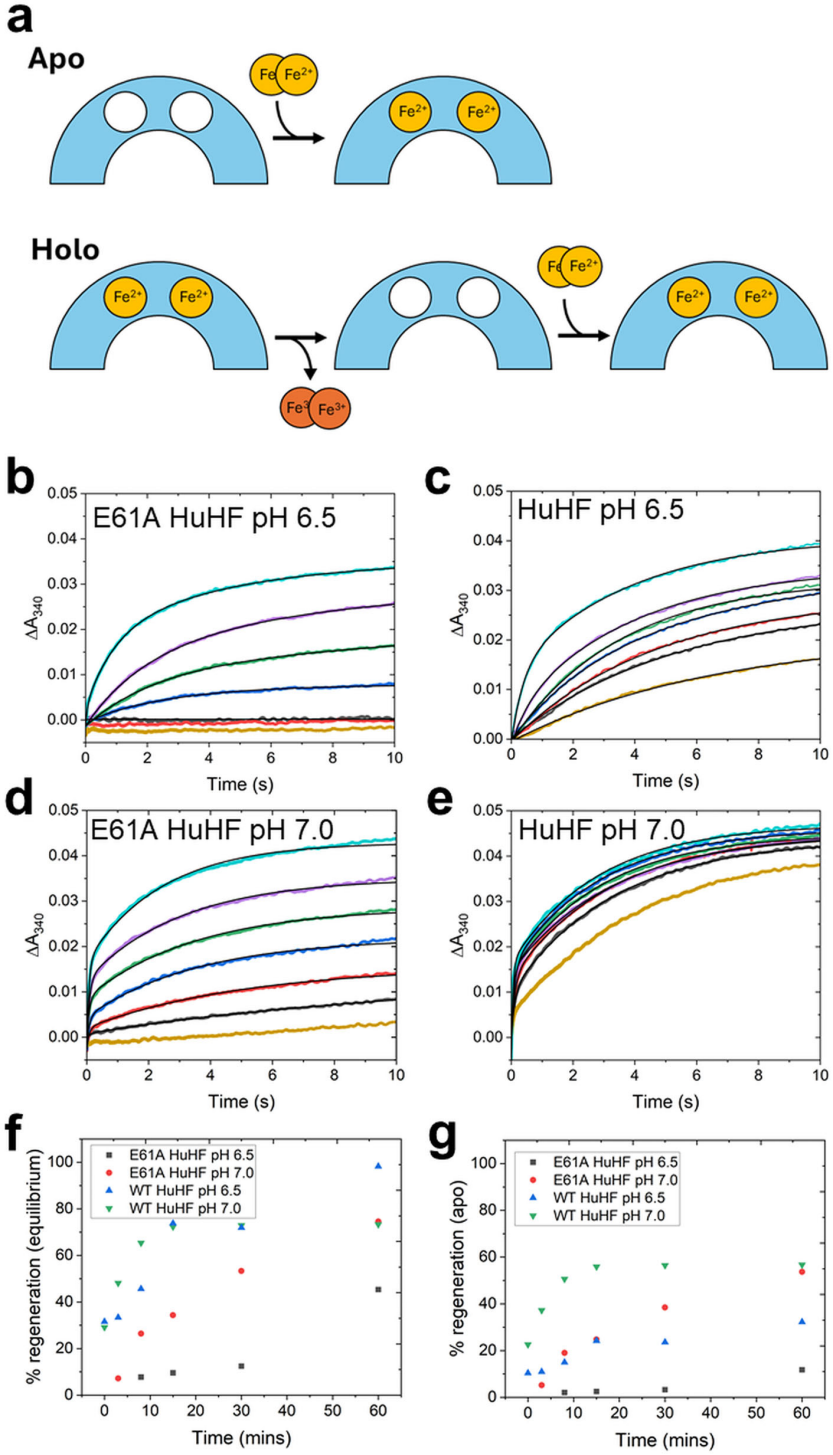
Exit of Fe^3+^ from the FoC and regeneration of apo form. (a) Schematic of Fe^2+^ binding to the apo FoC (top panel) or holo FoC (bottom panel) in ferritin H‐chain subunits. In the presence of O_2_, the Fe^2+^ ions are oxidised to Fe^3+^ to form a metastable di‐Fe^3+^ centre from which Fe^3+^ is lost to regenerate the apo form. (b–e) Regeneration of rapid iron oxidation phase measured by change in absorbance at 340 nm at 25°C following mixing of equal volumes of 48 equivalents of ferrous ammonium sulfate in 1 mM HCl and 1 µM E61A HuHF (b and d) and wild‐type HuHF (c and e) in 100 mM MES pH 6.5 (b and c) or 100 mM MOPS pH 7.0 (d and e). Proteins contained 200 equivalents of iron that were allowed to oxidise and then incubated for the times indicated in parts f and g of the figure, or overnight (corresponding to the largest absorbance change) prior to the addition of 48 Fe^2+^ ions. (f and g) Percentage of rapid oxidation activity regenerated at the indicated incubation time point relative to the equilibrium FoC state (f) and the apo FoC in E61A and wild‐type HuHF (g) at pH 6.5 and pH 7.0.

For wild‐type HuHF, some rapid Fe^2+^ oxidation activity was apparent at short incubation times, as shown in Figure 8, with regeneration occurring more rapidly at pH 7.0 (Figure 8e) than at pH 6.5 (Figure 8c). This suggests that the transfer of Fe^3+^ out of the FoC sites occurs more readily at higher pH, possibly because of an increasingly negative net charge favouring transfer. Recovery of rapid oxidation of the E61A variant was observed only at a very low level, even after 1 h at pH 6.5 (Figure 8b) and a significant effect was also observed at pH 7.0 (Figure 8d).

The extent of regeneration of the FoC was quantified by fitting the data using biexponential functions (Figure 8). As above, the biexponential function describes the rapid and slow phases; here, the amplitude associated with the rapid phase at ΔA_340 nm_ describes the extent to which apo FoCs were present at that incubation time. After an incubation period of 16 h (the overnight spectra in Figure 8), it was assumed that the FoC was at an equilibrium state between occupied and unoccupied states, meaning that longer incubation would not be expected to lead to further regeneration of apo sites. A comparison of amplitudes of the rapid phase following overnight incubation with those observed for the apo protein following addition of 48 Fe^2+^ ions (i.e., the maximum amplitude observed when only apo FoCs are present), as shown in Figure 7, revealed percentage regenerations of 32% and 75% for wild‐type HuHF, and 25% and 72% for E61A HuHF at pH 6.5 and 7.0, respectively. Thus, the equilibrium position was similar for both proteins and the replacement of Glu61 did not have a major impact. However, the rate at which the two proteins reached their equilibrium positions were very different.

Amplitudes of ΔA_340 nm_ corresponding to rapid FoC activity observed at incubation time periods over the first hour following the initial iron oxidation relative to that observed for the overnight incubation provided the percentage regeneration of the FoC activity relative to the equilibrium state amplitude (Figure 8e) and to the maximum amplitude (as observed for apo HuHF, Figure 8f) as a function of time. For both plots, E61A HuHF exhibited a significantly lower percentage regeneration rate compared to the wild‐type protein at both pH 6.5 and pH 7.0.

These results are similar to those observed for FtMt and its E61A variant [18]. For FtMt, it was concluded, in conjunction with the loss of iron binding on the inner surface, that Glu61 plays a key role in the transfer of Fe^3+^ from the FoC following oxidation. In the case of FtMt, Glu61 was also found to be essential for the observation of the nascent mineral core [18]. Similarly, for HuHF, Glu61 is centrally involved in the transfer of Fe^3+^ from the FoC following oxidation of Fe^2+^.

### 2.7 Conclusions and Perspective

The data presented here provide novel insight into the mechanism of Fe^2+^ oxidation and mineralisation in HuHF. Previously (Fe3 and Fe4) and newly (for HuHF) characterised (Fe5) near‐FoC iron‐binding sites are demonstrated to bind iron only after oxidation of Fe^2+^ at the FoC, that is, they are Fe^3+^‐binding sites. Each of them involves Glu61 as a coordinating residue, with the sidechain of Glu61 exhibiting significant flexibility in adopting one of two confirmations that suggest a mechanism by which Fe^3+^ exits the FoC and is transported to near‐FoC sites. A similar conformational flexibility of Glu61 was reported more than 30 years ago for a Tb^3+^‐bound form of HuHF (the first HuHF high‐resolution study) [21]. These structural data appear to map out a route by which, post‐oxidation, Fe^3+^ is shuttled away from the FoC to the inner surface of the HuHF subunit. Consistent with this, binding of Fe^3+^ at sites Fe3, Fe4 and Fe5 was not observed in structures of the E61A HuHF variant.

**FIGURE 9:**
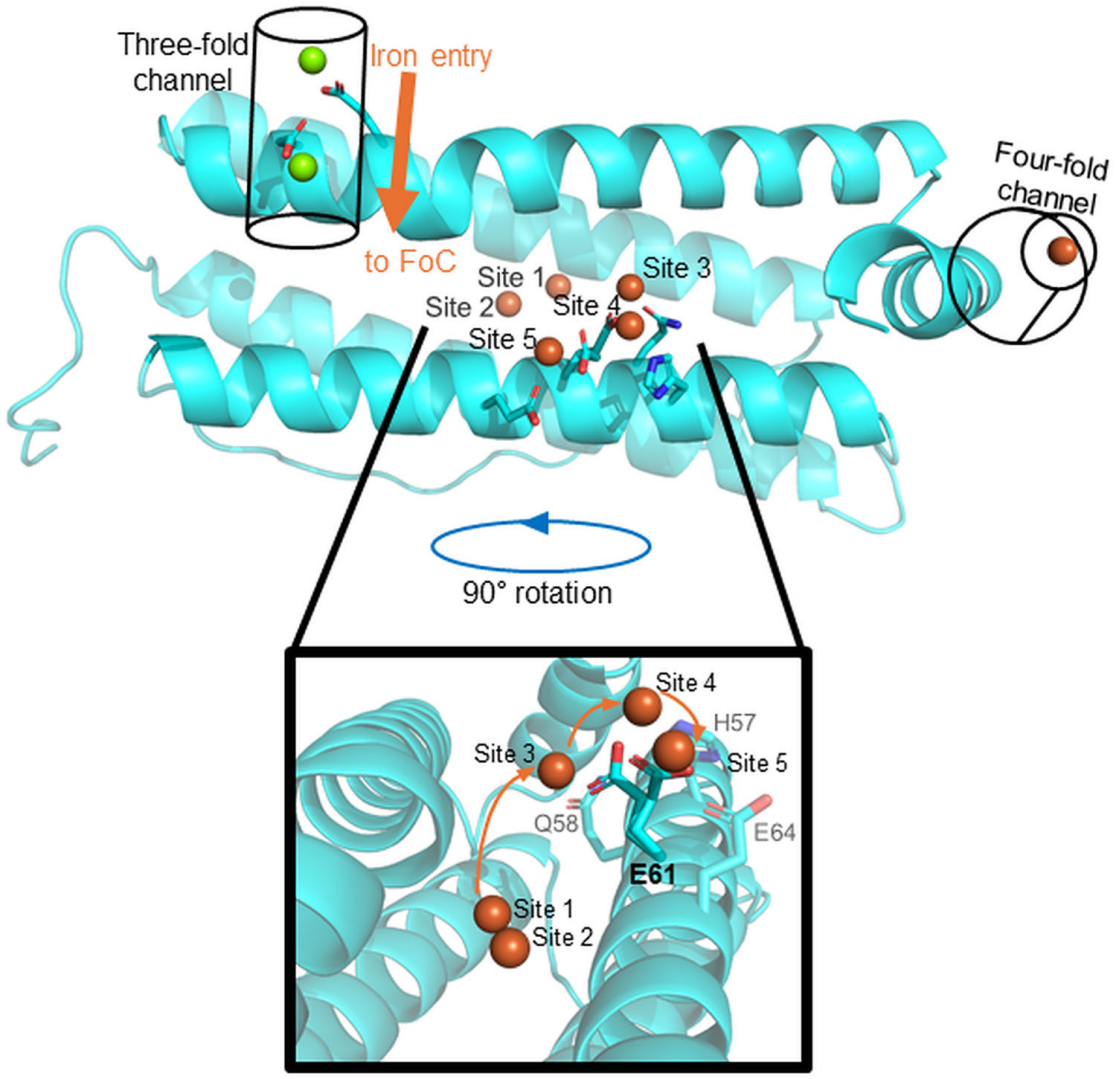
Proposed mechanism of iron entry into and exit from the FoC of HuHF. Data presented here indicate that the Glu61 sidechain is highly flexible and plays a key role in facilitating iron transfer. Image made in PyMOL. Note that the structure of HuHF with Fe^2+^ under anaerobic conditions demonstrated that, of the FoC/near‐FoC sites, only the FoC sites Fe1 and Fe2 were occupied, in agreement with a previous description [16], supporting the mechanistic scheme of Figure 1, which is distinct from those involving three Fe^2+^ sites at/close to the FoC [5]. Post‐Fe^2+^ oxidation, Fe^3+^ exits the FoC by transfer to site Fe3, assisted by the flexibility of the Glu61 sidechain. From there, the Fe^3+^ migrates further to site Fe4 and eventually to site Fe5, in each case coordinated by Glu61.

Arising from the time‐dependent oxidation structural data is a model, summarised in Figure 9, that is, entirely supported by the kinetic data. The latter shows that Glu61 plays no detectable role in Fe^2+^ binding or oxidation at the FoC but is of central importance for the transfer of Fe^3+^ out of the FoC. This is evidenced by the impact of substituting Glu61 both on the rate of mineralisation, which is rate‐limited by the transfer of Fe^3+^ out of the FoC, and on the rate of recovery of the apo form of the HuHF FoC, which is also limited by the transfer of Fe^3+^ out of the FoC. These data are consistent with early kinetic studies of site‐directed variants of HuHF suggesting that Glu61 is important for mineralisation [19].

The model presented here should be considered in the context of the model of Fe^3+^ transfer that resulted from an NMR study of the effect of Fe^2+^ oxidation on resonances arising from residues of the H’‐chain of *R. catesbeiana* [27]. That report suggested that Fe^3+^ ions migrate down the long axis of the inside of the four helical bundle of the H’‐subunit towards the four‐fold channels from where they enter the central cavity. We note that the near‐FoC iron sites of the *R. catesbeiana* H’‐chain exhibit some variation compared to those of HuHF [14] and that resonances due to residues associated with the near‐FoC sites identified by crystallography [14] were not amongst those that could be assigned. Thus, it is somewhat difficult to compare the data. Nevertheless, the two models are possibly consistent. The earlier NMR study, which was pioneering in demonstrating how difficulties associated with large, slow‐tumbling proteins could be overcome, provided a lower‐resolution but longer‐range view of the movement of Fe^3+^ away from the vicinity of the FoC, which the data reported here do not address. The NMR data are further discussed below.

Glu61 was shown recently to play a central role in the nucleation of the mineral core of FtMt and in the transfer of Fe^3+^ out of the FoC to the inner surface nucleation site [18]. Hence, there are several parallels between the roles of Glu61 in HuHF and FtMt. Perhaps the most important difference, however, is that HuHF is not the site of nucleation, whereas FtMt (a natural homopolymer) is. In cytosolic animal ferritins, which are heteropolymers of H‐ and L‐chain subunits, the site of nucleation is located on the inner surface of the L‐chain. Perhaps it is not surprising that this site involves the equivalent of Glu61 in L‐chain, in addition to residues Glu60 and Glu64 [6]. We propose that Glu61 of HuHF plays a crucial role in natural heteropolymer ferritins because it facilitates the transfer of Fe^3+^ ions through a series of sites towards the inner protein surface where they are subsequently shuttled (possibly from site Fe5) to the L‐chain nucleation site (or the growing mineral core surface).

Nevertheless, homopolymeric HuHF can mineralise iron. The data presented here and previously [18] showed that for HuHF nucleation does not occur at Glu61 as it does for FtMt [18]. This raises the question, then, of where it does occur. The NMR study of Turano et al. [27] indicated that the four‐fold channels appear to play a role in nucleation in the *R. catesbeiana* H’‐chain homopolymer, and this may also be the case for HuHF. The challenge now is to apply high‐resolution x‐ray crystallography and solution kinetic methods to follow Fe^2+^ oxidation and iron core nucleation in physiological heteropolymers of H‐ and L‐chains.

## Author Contributions

**Zinnia Bugg**: conceptualization, investigation, formal analysis, writing – original draft and data curation. **Justin M. Bradley**: conceptualization, supervision and writing – review and editing. **Andrew M. Hemmings**: investigation and supervision. **Nick E. Le Brun**: conceptualization, funding acquisition, supervision, writing – original draft and writing – review and editing.

## Conflicts of Interest

The authors declare that there are no competing interests associated with the manuscript.

## Supporting information




**Supporting File 1**: anie72772‐sup‐0001‐SuppMat.pdf.

## Data Availability

All data supporting the conclusions of this study are available within the paper and its Supporting Information or source data files. Final coordinates and structure factors were deposited in the Protein Data Bank (https://www.rcsb.org) with accession codes 28KA (HuHF anaerobic), 28KC (HuHF 2 min O_2_), 28LZ (HuHF 20 min O_2_), 28KB (HuHF 3 h O_2_), 28JY (E61A HuHF anaerobic), 28JZ (E61A HuHF 1 h O_2_).
