# Supplementary material for: Ferritin Iron Mineralisation: Route of Fe3+ Transfer From the Ferroxidase Centre to the Inner Cavity of Human H‐Chain Ferritin
Source: Angew Chem Int Ed Engl. 2026 May 30;65(31):e1203843. doi: 10.1002/anie.1203843 (PMC13411386; doi:10.1002/anie.1203843)
Supplement: Supplementary file 1 — Supporting File 1: anie72772‐sup‐0001‐SuppMat.pdf. [file ANIE-65-e1203843-s001.pdf]

Supporting Information

Ferritin Iron Mineralisation: Route of Fe^{3+} Transfer from the Ferroxidase Centre to the Inner Cavity of Human H-chain ferritin.

Zinnia Bugg, Justin M. Bradley, Andrew M. Hemmings and Nick E. Le Brun

Materials and Methods

Protein over-production and purification. Plasmids encoding HuHF and E61A variant proteins based on the pET21a expression vector were purchased from Genscript (Netherlands). Proteins were expressed using *Escherichia coli* strain BL21(DE3) as previously described [1]. Briefly, inoculated cultures were grown in LB media containing ampicillin at 100 µg mL^{-1} until the optical density at 600 nm was in the range 0.6-0.8. Expression was induced by addition of 100 µM. isopropyl β–d-1 thiogalactopyranoside (IPTG) and cultures incubated for a further 20 hr at 30 ˚C with shaking at 90 rpm prior to harvesting. Cell pellets were resuspended in 20 mM HEPES, 100 mM KCl, 0.1 mM EDTA, pH 7.8 (buffer A), disrupted by sonication and the resulting lysate heated at 65 ˚C for 15 min to remove thermally unstable proteins. Ferritin was precipitated using ammonium sulfate at a concentration of 0.55 g mL^{-1} and subsequently redissolved in buffer A and dialyzed to remove ammonium sulfate. The resulting solution was applied to a HiPrep 26/60 Sephacryl S300HR size exclusion column (Cytiva) and ferritin-containing fractions combined and applied to a HiTrap Q FF anion exchange column (Cytiva) to remove contaminating DNA. Protein solutions were loaded in buffer A and eluted by stepping to 30% buffer B (20 mM HEPES, 100 mM KCl, 1 M NaCl, 0.1 mM EDTA, pH 7.8). Any iron bound to the ferritin proteins was removed using the method of Bauminger *et al* [2]. Protein concentration was determined by absorbance assuming $\varepsilon_{280\ nm}$ = 4.08 ×10^5 M^{-1} cm^{-1} for the 24meric protein cage [3].

Crystallography of iron-loaded E61A and wild-type HuHF. Protein solutions (10 mg mL^{-1} for wild-type HuHF and 12 mg mL^{-1} for E61A HuHF) were exchanged into 20 mM MES, pH 6.5 and 2 µL drops were mixed in equal volume with well solution (100 mM bicine 100 mM NaCl 2 M $MgCl_2$ 60 mM $FeCl_2$ 3 mM NaN_3 pH 9.0) in a N_2-filled chamber (Belle technology, [O_2] < 10 ppm) and equilibrated in sitting drops by vapour diffusion against 200 µL of the well solution at 16 °C. Crystals with bi-pyramidal symmetry appeared within 24 hr and grew to 100 - 200 µm within two weeks. Crystals were transferred to cryoprotectant consisting of aerobic (air-saturated) well solution with 30% (v/v) glycerol and 2.2 M $MgCl_2$ for O_2 exposure before flash freezing in liquid N_2. Diffraction data were collected at the Diamond Light Source (Didcot, UK) on beamline i24 or i04 with a wavelength dependent on the experimental aim: at high energy (0.999 or 0.9763 Å) for maximum resolution, at the peak of the iron K absorption (1.7395 Å) to maximise the anomalous scattering intensity, and low energy of the Fe absorption (1.75 Å) to aid with metal ion identification. Protein structures were solved by molecular replacement using apo HuHF (PDB entry 5N27 [4]) as the search model. All structures were solved to Rfree < 25%. The asymmetric unit of all the datasets of the crystals consists of one subunit of the HuHF, consistent with the literature structures of apo and iron-loaded HuHF. All data was indexed and processed using XDS and Aimless as part of the automatic xia2 pipeline [5]. All data was symmetry-indexed to F432 space group, so have one monomer in the unit cell. Structures were solved by molecular replacement with phenix.phaser MR [6] using PDB entry 5N27 (1.7 Å resolution structure of wild-type apo HuHF) as the search model [4]. Structure refinement employed iterative cycles of refinement using phenix.refine and WinCOOT for manual correction [7]. Metals placement was performed by reference to Bijvoet-difference Fourier maps. No metal coordination restraints were applied to the metal sites. Metal site occupancies were manually adjusted to be refined so that the anisotropic temperature B factor fell within ± 15% of the B factors for surrounding atoms. Statistics relating to data collection and struture solution and refinement, and for data used for calculation of anomalous scattering density maps, are shown in Table S1 and S2).

Absorbance-monitored kinetic studies. Protein-catalysed Fe^{2+} oxidation/mineralisation activity was monitored via the increase in absorbance at 340 nm following addition of 400 equivalents of Fe^{2+} to apo ferritin at 25 ˚C. Aerobic ferrous ammonium sulfate dissolved in 1 mM HCl (to inhibit Fe^{2+} oxidation) was added to a final concentration of 200 µM to an aerobic 0.5 µM solution of protein (wild-type or E61A HuHF) in 100 mM MES pH 6.5 in a 1 cm pathlength cuvette. Initial rates of reaction were calculated from the slope of the linear region of a plot of absorbance at 340 nm vs. time [1].

The initial rapid oxidation reaction at the FoC could not be adequately following using the above conventional mixing experiment. Thus, it was measured by stopped-flow rapid mixing using an Applied Photophysics Bio-Sequential DX.17MV spectrophotometer with a 1 cm path length observation cell. Equal volumes of aerobic 1 µM apo protein in 100 mM MES pH 6.5 or 100 mM MOPS pH 7.0 and aerobic solutions of 6, 12, 18, 24, 30, 36, 42, 48, 60, 72 or 96 µM ferrous ammonium sulfate in 1 mM HCl were mixed. The time dependences of absorbance increases at 340 nm were fitted using a biexponential function (Equation 1), representing rapid (r) and slower (s) components, using OriginPro 2024b (OriginLab):

$$\Delta A_{340}(t) = \Delta A_{340}^{(tot)} - \Delta A_{340}^{r} e^{-k_r t} - \Delta A_{340}^{s} e^{-k_s t} \qquad \text{Equation 1}$$

Following oxidation of Fe^{2+} at the FoCs of HuHF, Fe^{3+} exits the FoC and enters the cavity where it is incorporated into a growing mineral core. The rate at which Fe^{3+} exits the FoC is difficult to measure directly but can be estimated by instead measuring the rate at which rapid Fe^{2+} oxidation (i.e. the rapid phase component) at the FoC recovers, because rapid oxidation can only occur once Fe^{3+} has exited the active site, i.e. it is a characteristic of apo protein. Aerobic HuHF (or variant) protein solution (1 µM at either pH 6.5 or 7.0) was incubated with aerobic 200 µM Fe^{2+} at 25 ˚C until the absorbance at 340 nm became invariant with time, indicating full oxidation of Fe^{2+} to Fe^{3+}. Samples were then mixed with an equal volume of 48 µM Fe^{2+} in 1 mM HCl either immediately or following a further period of incubation of 3, 8, 15, 60 min or overnight at 25 °C. Absorbance at 340 nm as a function of time was measured (by stopped-flow as above) at 25 °C.

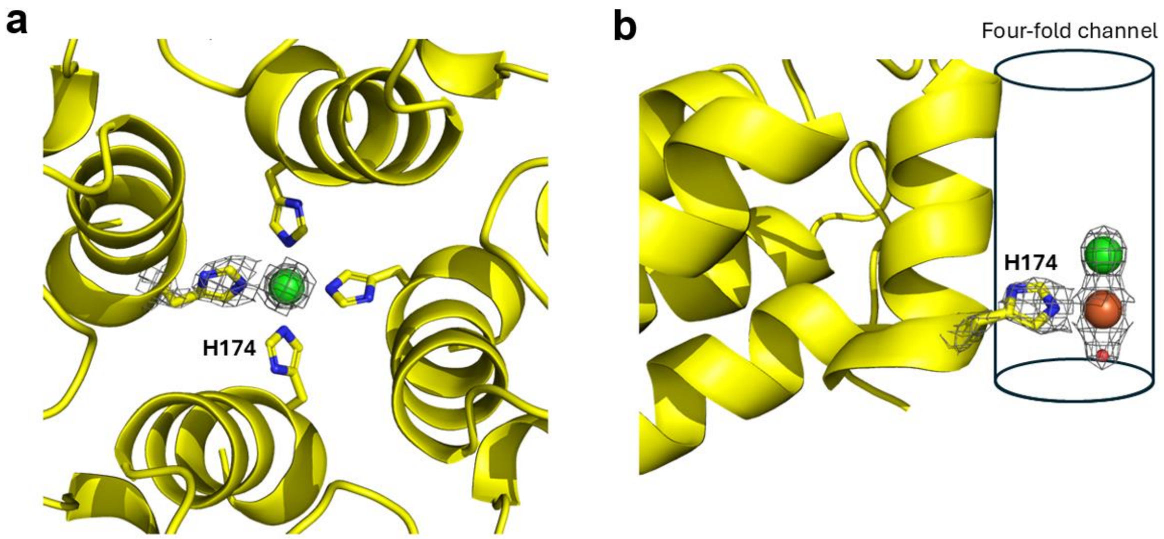

Figure S1. The four-fold channel of anaerobic iron-loaded HuHF, viewed (**a**) down the channel axis, and (**b**) rotated 90° to the view in (A). Grey mesh shows the Sigma-A weighted Fourier (2mFo-DFc) map contoured at 1 σ for ions and the associated residue His174. Iron (orange sphere) is shown coordinated to water (red sphere), chloride (dark green sphere) and His174. Images made in PyMOL.

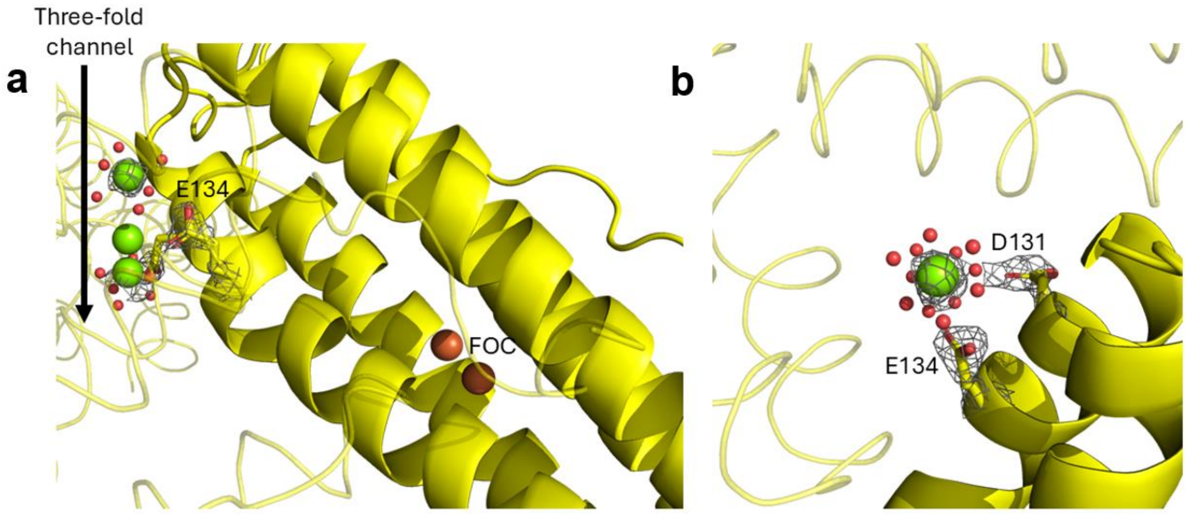

Figure S2. The three-fold channel of anaerobic iron-loaded HuHF, viewed (**a**) side on to the channel long axis, and (**b**) down (along) the channel axis. Grey mesh shows the Sigma-A weighted Fourier (2mFo-DFc) map contoured at 1 σ for ions and the associated residues Asp131 and Glu134. Magnesium ions are shown by green spheres and waters by red spheres. Iron ions at the FoC are indicated by orange spheres. Images made in PyMOL.

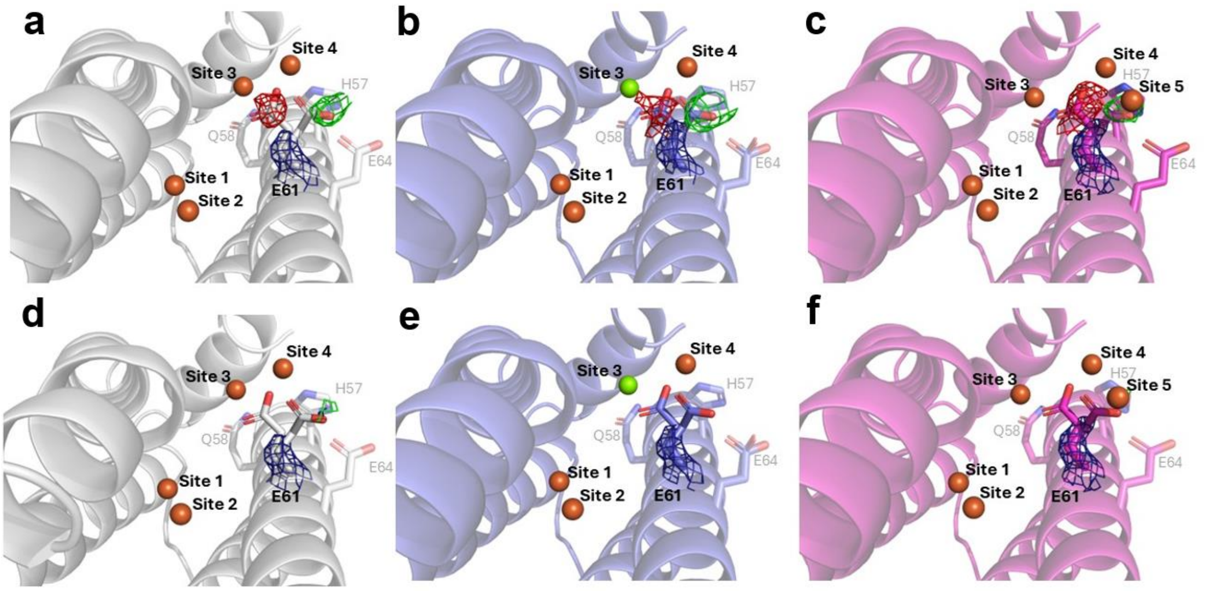

Figure S3. Iron-loaded HuHF crystal structures with O_2 soak times of 2 min (**a** and **d**), 20 min (**b** and **e**) and 3 hr (**c** and **f**) viewed along the subunit bundle with the inner surface of the ferritin on the right. The structures in (a) – (c) were refined without the second Glu61 conformer, and (d) – (f) were refined with this conformer included. The resulting 2mFo-DFc maps are shown in blue (contoured to 1 σ) along with the Fo-Fc map (positive shown in green to +3 σ and negative shown in red to -3 σ). Iron atoms are shown as orange spheres and magnesium are shown in green. Images were made in PyMOL.

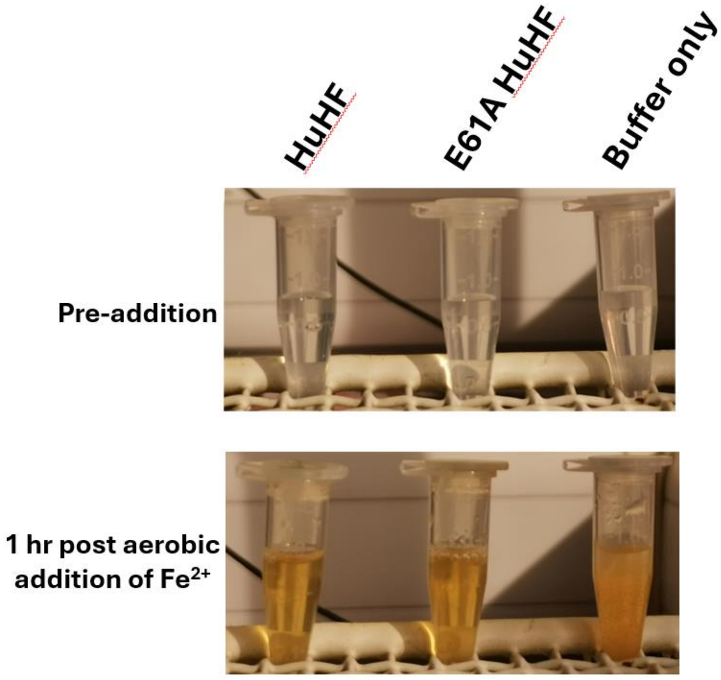

Figure S4. Images of ferritin protein and buffer-only solutions pre- and post-addition of Fe^{2+} ions under aerobic conditions. Proteins (as indicated) were at 2.5 μM concentration in 100 mM MOPS, pH 7. The buffer-only solution was 100 mM MOPS, pH 7. Images show the samples pre- and 1 hour post- aerobic addition of 2 mM Fe^{2+}. This represents twice the level of Fe^{2+} added in experiments described in Fig. 6, chosen to make it easier to see the differences between the before and after and the protein and buffer-only samples.

Table S1. X-ray data collection and refinement statistics*.

Protein	HuHF anaerobic	HuHF 2 min	HuHF 20 min	HuHF 3 hour	E61A HuHF anaerobic	E61A HuHF 1 hour
PDB code	28KA	28KC	28LZ	28KB	28JY	28JZ
Wavelength (Å)	0.9999	0.9763	0.9763	0.9999	0.9999	0.9999
Resolution range (Å)	28.93 - 2.106 (2.181 - 2.106)	91.47 - 1.94 (2.009 - 1.94)	91.38 - 1.941 (2.01 - 1.941)	46.04 - 1.83 (1.896 - 1.83)	55.09 - 1.6 (1.657 - 1.6)	29.05 - 2.41 (2.496 - 2.41)
Space group	F 4 3 2	F 4 3 2	F 4 3 2	F 4 3 2	F 4 3 2	F 4 3 2
Unit cell	182.958 182.958 182.958 90 90 90	182.94 182.94 182.94 90 90 90	182.76 182.76 182.76 90 90 90	184.16 184.16 184.16 90 90 90	182.7 182.7 182.7 90 90 90	183.718 183.718 183.718 90 90 90
Total reflections	31515 (3063)	38141 (2129)	37937 (2100)	48327 (4690)	64922 (6884)	21554 (2082)
Unique reflections	15758 (1532)	19352 (1322)	19226 (1293)	24165 (2345)	34974 (3444)	10777 (1041)
Multiplicity	2.0 (2.0)	2.0 (1.6)	2.0 (1.6)	2.0 (2.0)	2.0 (2.0)	2.0 (2.0)
Completeness (%)	99.86 (99.61)	96.56 (66.34)	96.40 (66.17)	99.97 (99.91)	99.95 (99.88)	99.86 (100.00)
Mean I/sigma(I)	56.52 (7.82)	28.65 (0.60)	39.05 (0.91)	18.97 (0.71)	14.75 (0.76)	42.1 (8.90)
Wilson B-factor	26.39	31.18	31.56	35.45	19.43	38.67
R-merge	0.01379 (0.08272)	0.035 (0.6772)	0.03351 (0.474)	0.02426 (0.9149)	0.03663 (0.9721)	0.01252 (0.06707)
R-meas	0.01951 (0.117)	0.0495 (0.9577)	0.04739 (0.6704)	0.03431 (1.294)	0.05181 (1.375)	0.0177 (0.09485)
R-pim	0.01379 (0.08272)	0.035 (0.6772)	0.03351 (0.474)	0.02426 (0.9149)	0.03663 (0.9721)	0.01252 (0.06707)
CC1/2	1 (0.979)	0.998 (0.49)	0.998 (0.687)	1 (0.44)	0.999 (0.397)	1 (0.987)
CC*	1 (0.995)	1 (0.811)	1 (0.902)	1 (0.782)	1 (0.754)	1 (0.997)
Reflections used in refinement	15757 (153)	19333 (1305)	19221 (1289)	24162 (2343)	34963 (3440)	10777 (1041)
Reflections used for R-free	2000 (194)	945 (61)	935 (56)	1267 (138)	1805 (174)	1079 (105)
R-work	0.1543 (0.1533)	0.1681 (0.3442)	0.1678 (0.2925)	0.1920 (0.3959)	0.1811 (0.3284)	0.1853 (0.2087)
R-free	0.1915 (0.2185)	0.1924 (0.3692)	0.1948 (0.3633)	0.2278 (0.4078)	0.1981 (0.3309)	0.2389 (0.3007)
CC(work)	0.960 (0.954)	0.960 (0.608)	0.961 (0.742)	0.948 (0.639)	0.957 (0.641)	0.946 (0.910)
CC(free)	0.946 (0.898)	0.955 (0.522)	0.955 (0.567)	0.906 (0.749)	0.944 (0.606)	0.912 (0.753)
Number of non-hydrogen atoms	1645	1717	1723	1597	1731	1463

**macromolecules**	1435	1543	1529	1443	1415	1399
**ligands**	14	12	15	16	14	9
**solvent**	196	162	179	138	302	55
**Protein residues**	174	174	174	174	173	171
**RMS(bonds)**	0.007	0.008	0.008	0.008	0.006	0.007
**RMS(angles)**	0.82	0.96	0.98	0.95	0.79	0.82
**Ramachandran favoured (%)**	98.26	98.84	98.26	98.84	98.25	97.63
**Ramachandran allowed (%)**	1.74	1.16	1.74	1.16	1.75	2.37
**Ramachandran outliers (%)**	0	0	0	0	0	0
**Rotamer outliers (%)**	0.65	1.18	2.40	0.64	0	0
**Clashscore**	2.12	1.63	2.96	4.58	2.87	5.81
**Average B-factor ($Å^2$)**	28.29	33.71	34.22	37.14	22.69	42.07
**macromolecules**	27.08	32.66	33.03	36.18	20.6	41.93
**ligands**	35.17	43.45	44.94	45.29	25.77	42.59
**solvent**	36.67	42.98	43.52	46.23	32.34	45.59

*Data in brackets refers to highest resolution shell

Table S2. Statistics for datasets used for calculation of Bijvoet-difference Fourier maps.

Protein	HuHF anaerobic	HuHF 2 min	HuHF 20 min	HuHF 3 hour	E61A HuHF anaerobic	E61A HuHF 1 hour
**Beamline**	DLS i24	DLS i04	DLS i04	DLS i24	DLS i24	DLS i24
**Wavelength (Å)**	1.75	1.75	1.75	1.75	1.75	1.75
**Resolution range (Å)**	28.93 - 2.106	91.46 - 2.0	91.38 - 1.938	55.19 - 1.99	92.00 - 2.14	64.71 - 2.05
**Total reflections**	31515	35369	37947	2320183	2083280	34061
**Unique reflections**	15758	17730	19235	18609	15387	17034
**Multiplicity**	2.0	2.0	2.0	124.7	135.39	2.0
**Completeness (%)**	99.86	96.54	96.03	96.33	100.00	99.62
**Anomalous multiplicity**	71	75.3	69.3	68.1	74.44	39.3
**Anomalous completeness (%)**	99	96.53	93.03	100	100.00	100
**<I>**	47	12	22	37	5.7	15
**<I/σI>**	56.52	25.83	39.03	16.55	1.5	38.03
**Rmerge**	0.01379	0.03649	0.03351	0.2481	6.4279	0.03362
**Rmeas**	0.01951	0.05161	0.04739	0.249	6.452	0.04755
**Rpim**	0.01379	0.03649	0.03351	0.02128	0.55	0.03362
**CC(1/2)**	1	0.998	0.998	0.999	0.9428	0.998
**CC(anom)**	0.534	0.102	0.341	0.205	0.065	0.202
**Anomalous signal resolution limit (Å)[&]**	2.59	2.7	2.3	3.07	3.59	1.77

[&] Resolution limit of the anomalous signal calculated by Aimless [8].

References

[1] J. M. Bradley, Z. Bugg, G. R. Moore, A. M. Hemmings, N. E. Le Brun, *J. Am. Chem. Soc.* **2025**, *147*, 13699–13710.

[2] E. R. Bauminger, P. M. Harrison, D. Hechel, I. Nowik, A. Treffry, *Biochim. Biophys. Acta* **1991**, *1118*, 48–58.

[3] F. Bou-Abdallah, P. Santambrogio, S. Levi, P. Arosio, N. D. Chasteen, *J. Mol. Biol.* **2005**, *347*, 543–554.

[4] G. Ferraro, S. Ciambellotti, L. Messori, A. Merlino, *Inorg. Chem.* **2017**, *56*, 9064–9070.

[5] G. Winter, *J. Appl. Crystallogr.* **2010**, *43*, 186–190.

[6] P. V. Afonine, R. W. Grosse-Kunstleve, N. Echols, J. J. Headd, N. W. Moriarty, M. Mustyakimov, T. C. Terwilliger, A. Urzhumtsev, P. H. Zwart, P. D. Adams, *Acta Crystallogr. D Struct. Biol.* **2012**, *68*, 352–367.

[7] P. Emsley, B. Lohkamp, W. G. Scott, K. Cowtan, *Acta Crystallogr. D Biol. Crystallogr.* **2010**, *66*, 486–501.

[8] P. R. Evans, G. N. Murshudov, *Acta Crystallogr. D Biol. Crystallogr.* **2013**, *69*, 1204–1214.
